# A century of high elevation ecosystem change in the Canadian Rocky Mountains

**DOI:** 10.1038/s41598-020-66277-2

**Published:** 2020-06-16

**Authors:** Andrew Trant, Eric Higgs, Brian M. Starzomski

**Affiliations:** 1 School of Environment, Resources and Sustainability, University of Waterloo, Waterloo, ON Canada; 2 School of Environmental Studies, University of Victoria, Victoria, BC Canada

**Keywords:** Biodiversity, Biogeography, Climate-change ecology

## Abstract

Mountain ecosystems serve as sentinels of change, and those in the Canadian Rocky Mountains have undergone a pronounced shift over the past century. We present quantitative analyses of 81 high-resolution image pairs of systematic historic surveys and repeat photographs of Canadian Rocky Mountain habitats, measuring treeline advance, changes in tree density, and shifts in growth form from krummholz to trees. With a time-lapse of 68 to 125 years (mean 93.5 years) between image pairs, these photographs contain novel information about long-term ecological change across broad spatial scales. In the 197 linear km of mountain habitat over 5 degrees of latitude examined, we found evidence of treeline advance at 90/104 sites, increases in tree density at 93/104 sites, and many sites (79/95) showing detectable changes in the growth form of trees from krummholz to erect tree form. Using generalized linear mixed models, we found that treeline at higher altitudes and further north had a greater probability of advancing while regional climate factors in our model did not significantly explain our results. Historic references, such as those documented here, are invaluable for providing conservation targets and for contextualizing disturbance and broad scale ecosystem change.

## Introduction

Mountain ecosystems serve as sentinels of change. Understanding these changes is important for water and food security, forestry, biodiversity, and many forms of tourism and recreation^1,2^. The Canadian Rocky Mountains have experienced many of these shifts over the past century^3–5^. One aspect of mountain ecosystems that is often examined for change is the upper altitudinal limit of tree growth, referred to as the forest-alpine tundra ecotone, or treeline ecotone. The position of the treeline ecotone is generally considered to be controlled by a combination of temperature^6^, disturbance^7^, species interactions^8,9^, and topographic factors^10^. In many mountain ecosystems, especially in Europe, generations of people engaged in agriculture and livestock grazing have also dramatically influenced the position of the treeline^11^. A global metanalysis of the treelines found an upward or poleward shift in the position of the treeline over the past century^12^, which is the predicted response of the treeline to climate change. Thus, nearly half of the observed treelines examined in this study^12^ are not responding or are not responding in the expected direction. Variation in how treelines have been responding to climate change is attributed to species-specific responses^13^, herbivory^8,9^, or warming thresholds that have not yet been passed.

The methods of study available to examine treeline change will guide the temporal and spatial scale of the results. Dendroecology can provide high-resolution, species-specific data on treeline change that can extend back centuries but this approach often has spatial limitations (<1 km^2^) associated with the time-intensiveness of field sampling^13,14^. Remotely-sensed products can be used to examine treeline change at large spatial scales, far exceeding 1 km^2^, but lack species-specific information and with the wide-spread availability of satellite imagery only beginning in the early 1980s, these methods lack the temporal depth of other approaches^15,16^. In most instances, oral and traditional knowledge around treeline change is incomplete or restricted to areas of local interest, lacking broad spatial coverage^17^. Repeat photography, while often lacking species-specific information, can provide large spatial and temporal coverage and has been used successfully to document treeline and northern landscape change^18–21^.

In this study, we present quantitative analyses of 81 high-resolution image pairs of 104 treeline ecotones spanning approximately 100 years and 197 linear km to address the following research questions: 1) How much treeline advance, tree density change, and shifts from krummholz to tree growth forms has occurred in these mountain ecosystems over the last century?; and 2) To what extent can observed changes be explained by regional climate change, disturbance, and site factors (e.g. slope and altitude)? We will also explore the value of using these images as historical references for identifying boundaries and thresholds of change in mountainous environments.

## Materials and Methods

### Study area

**Figure 1:**
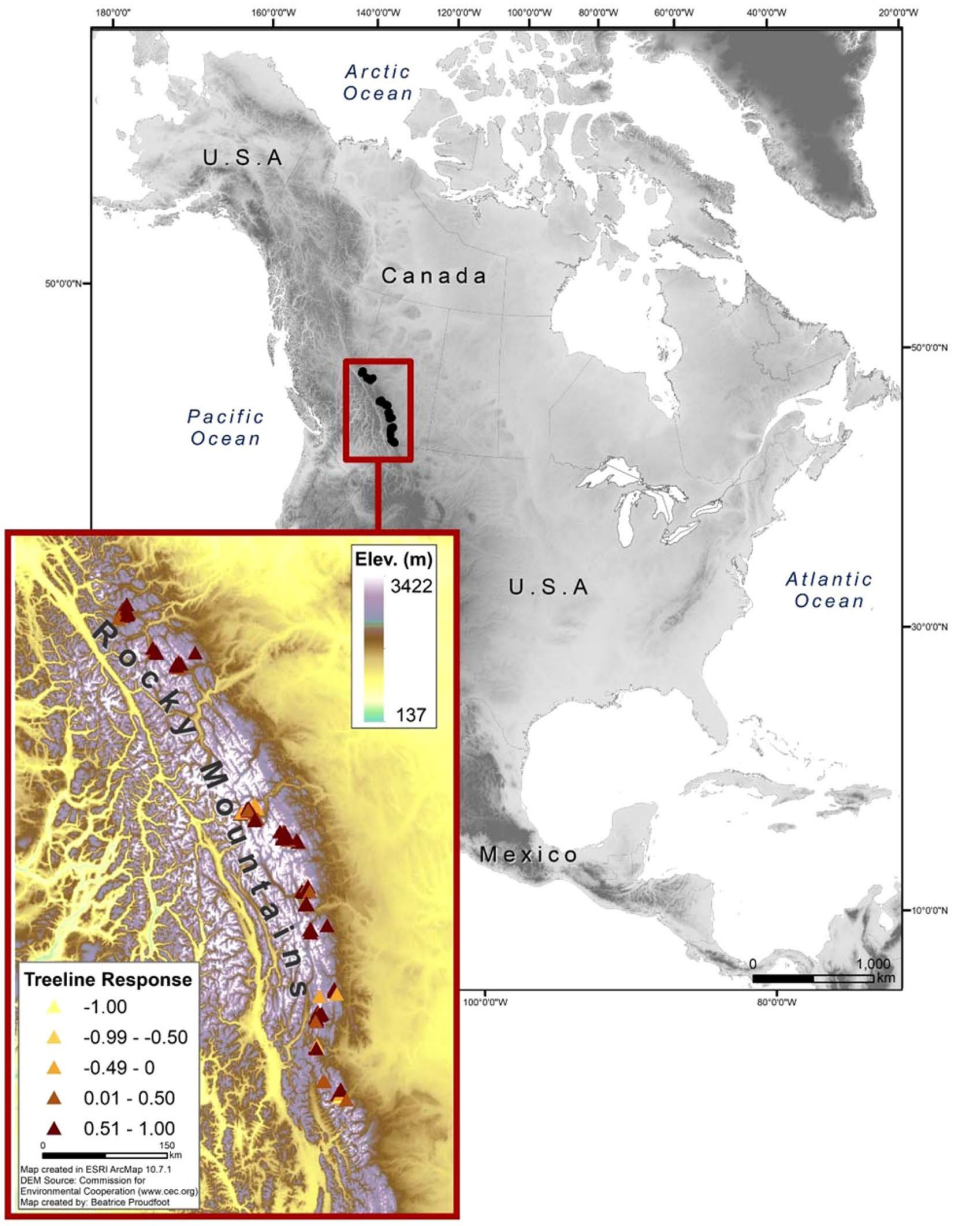
Map of study area showing the location of historic and repeat images (triangles) in the Canadian Mountain West. The colour of the triangles corresponds to the extent of change documented for each site as expressed as the consistency score averaged for each site with treelines having: increased (1), remained stable (0), or decreased (−1).

The study area for this research is the Canadian Rocky Mountains (Fig. 1). The Canadian Rocky Mountains comprise unprecedented ecological diversity with large contiguous regions that help maintain significant carnivore populations and numerous species of conservation concern^22^. A diversity of ecosystems can be found through this montane region from forested valley bottoms that transition into alpine tundra and rock dominated peaks at the higher elevations. The transition from forested to alpine-tundra plant communities, henceforth treeline ecotone, is dominated by subalpine fir (*Abies lasiocarpa* (Hooker) Nuttall), Engelmann spruce (*Picea engelmannii* Parry ex Engelmann), and subalpine larch (*Larix lyallii* Parlatore). Whitebark pine (*Pinus albicaulis* Engelm), has endangered status in Canada with a limited distribution across the treeline ecotone. Tree density generally decreases across the treeline ecotone with krummholz, defined here as shrub-like trees with prostrate growth forms, being common at the upper limit of where trees are found growing. Over the period of 1981–2010, the region has experienced average annual air temperatures of −0.14 ± 0.10 °C standard error (SE)(min = −2.1 °C, max = 2.6 °C) with summer average of 9.48 ± 0.11 °C (min = 7.5 °C, max = 12.2 °C) and winter average of −9.37 ± 0.11 °C (min = −11.4 °C, max = −6.4 °C). Mean annual precipitation values for the same period are 1022.39 ± 28.10 mm (min = 650 mm, max = 1828 mm), with winter and summer values being 224.08 ± 8.98 mm (min = 86 mm, max = 483 mm), and 297.81 ± 5.87 mm (min = 206 mm, max = 450 mm), respectively. Mean values presented here were generated from all study sites with the ClimateNA v5.10 software package based on methodology described by Wang *et al.*^23^.

The ecological and geological diversity of this region has been conserved in a number of National Parks (Banff, Jasper, Kootenay, Yoho, and Waterton Lakes), Provincial Parks (e.g. Mount Robson and Mount Assiniboine), and represents the core of the Yellowstone to Yukon (Y2Y) conservation and restoration initiative that focusses on landscape-scale connectivity. There has been a long history of Indigenous peoples living and harvesting resources throughout the Canadian Rocky Mountains - practices that continue today at least in various forms in spite of more than a century of colonial land-use change. These resource-use patterns are not expected to alter high elevation vegetation structure.

### Image collection

The Mountain Legacy Project contains a photographic database with over 120,000 historic images taken between 1888 and 1958 during cartographic surveys of the Canadian Mountain West by the Canadian federal and provincial government agencies. Over 8,000 of these scenes have been repeated and are freely available for the public to use^24^. From the Mountain Legacy Project collection, 81 image pairs from the Canadian Rocky Mountains were selected based on the presence of a treeline ecotone in both the historic and repeat images that are of suitable quality (i.e., in focus and without high contrast caused by shadows) and also that spanned the extent of the Canadian Rocky Mountains. Despite the large number of images available, surprisingly few met our aforementioned criteria. From these 81 image pairs, we analyzed 104 treeline ecotone sites.

### Quantifying treeline change

**Figure 2:**
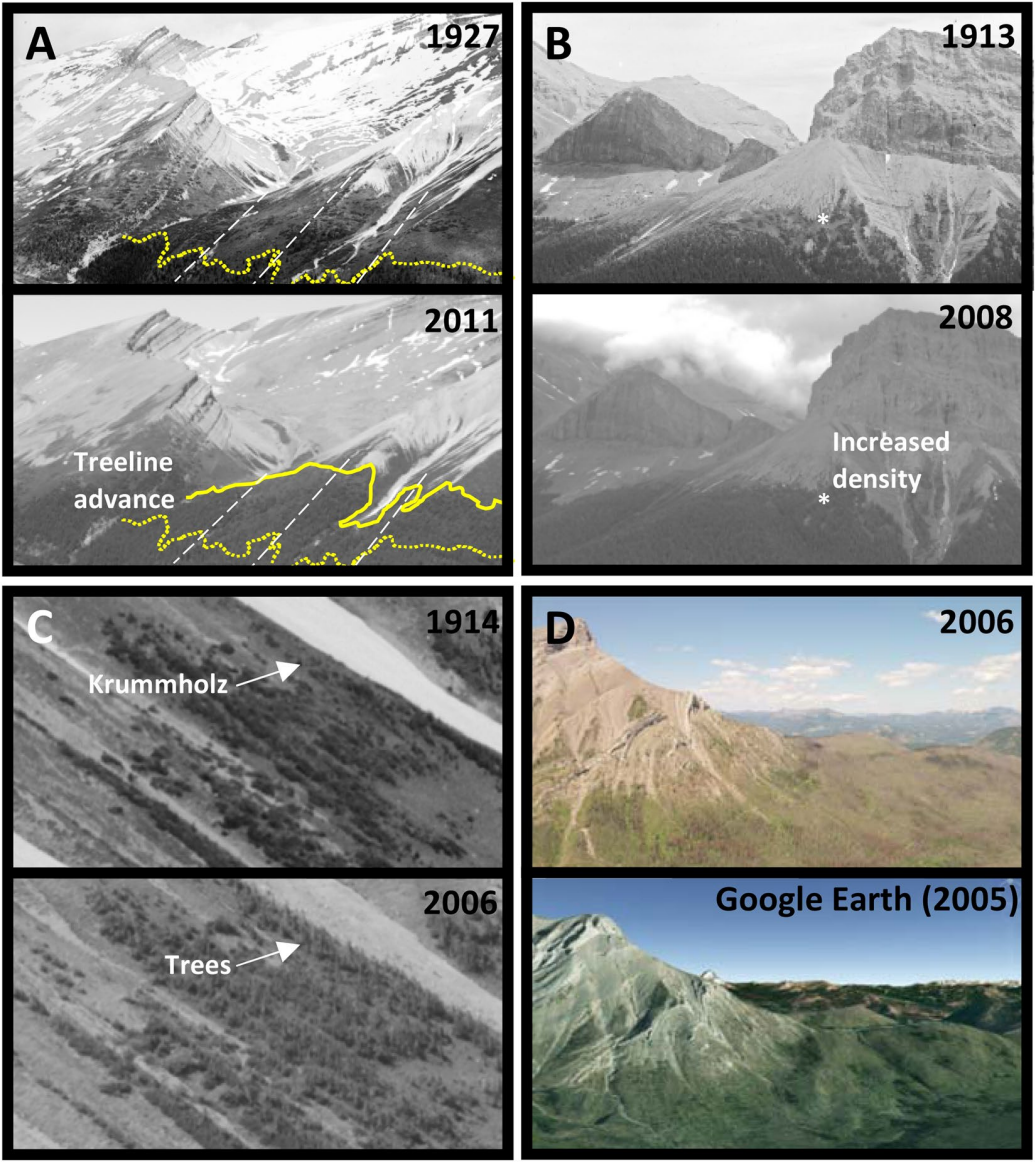
Historic and repeat photography examples of (**A**) treeline advance (yellow lines) with white lines showing demarcated segments used for analyses (image 38); (**B**) increases in tree density at the treeline ecotone with a specific location signified by the ‘*’ (image 2); and 3) shifts from krummholz to trees, and (**D**) example of repeat photograph and Google Earth digital elevation model (Image: Google, Maxar Technologies). Historic images courtesy of Library and Archives Canada. Repeat images courtesy of the Mountain Legacy Project (mountainlegacy.ca).

The historic and repeat images were analyzed using a Wacom Intuos Pro L tablet and custom Image Analysis Toolkit, software integrated into the Mountain Legacy Project website^25^, for delineating the treeline ecotone features directly onto the fully magnified image. The historic images were all captured using large format photography with glass plate negatives, resulting in black and white images^26^. To avoid any colour-informed bias, the repeat images were converted to greyscale prior to analyses. The treeline was delineated based on the altitudinal limit of where trees were growing with detectable vertical growth (i.e. leaders) when images were magnified to the maximum extent possible where individual stems could be discerned. This criterium was included to differentiate shrubs and krummholz from trees. The delineated treeline for the historic and repeat images were overlaid on both images with the length of each treeline then divided into four equal segments (Fig. 2A). For each segment, the following metrics were determined by assessing differences between the historic and repeat images:

1. ***Treeline advance and approximate distance of advance***. This was determined based on whether the position of the treeline had advanced (1), remained stable (0) or retreated (−1). A consistency score was than calculated for each treeline (e.g. 1 of 4 segments advanced = 0.25 [low consistency]; 4 of 4 segments advanced = 1 [high consistency]). The approximate distance that the treeline advanced or retreated (bins: 0 m, 5 [0.1–5 m], 25 [6–50 m], 75 [51–100 m], 250 [>100 m]) was also determined as the maximum for each segment and then analyzed using the mean of the four segments.
2. ***Change in tree density at the treeline ecotone***. In the upper 100 m limit of the treeline ecotone, we performed a visual assessment of each segment to determine if tree density had increased (1), remained stable (0), or decreased (−1) within each segment. A consistency score was then calculated for each treeline ecotone by taking the mean of the four segment values (Fig. 2B).
3. ***Shifts in growth form from krummholz to trees***. Krummholz patches in the treeline ecotone of historic images were compared to the repeat images to determine the initiation of visible leaders. These leaders signify a change from krummholz to trees, and were scored as detected (1) or not detected (0). A consistency score was then calculated for each treeline ecotone by taking the mean of the four segment values (Fig. 2C).

In each image, disturbance factors (insect outbreak and fire) at the treeline ecotone were recorded (*Disturbance*: 0 [absent], 1 [historic], or 2 [repeat]). We did not differentiate between disturbance types as we were not confident in making definitive determinations from historic and repeat images. Treeline form (*TreelineForm*: abrupt or diffuse) was also recorded for each treeline ecotone. Using a digital elevation model integrated into Google Earth, the slope was averaged from four transects parallel to the slope, equally spaced along the length of the treeline ecotone (*Slope*: degree), aspect (*Aspect*: warm [W, SW, S, SE] and cold [NW, N, NE, E]), and elevation of modern treeline (*Elevation*: in metres) were recorded (Fig. 2D). Google Earth was also used to determine the length, perpendicular to slope, of each treeline ecotone. Gridded climate data, at 1 km resolution, from 1901 to 2010 were generated with the ClimateNA v5.10 software package, based on methodology described by Wang *et al.*^23^. To compare the overall climatic regime for each site, we used the earliest available 30-year normal period, 1901–1930, representing climatic conditions of the historic images. The historical climate data were then compared to the 30-year normal period encompassing the repeat images (1981–2010). Given the climate variability across mountainous regions e.g.^27^, absolute values from climate data were not used (e.g. mean summer temperature from 1980–2010) but rather the magnitude of change between the historic and repeat images, which we assumed to be relevant. While we explored climate relationships using 30-year climate normal periods, it is possible that using different temporal scales (i.e., decadal) could offer interesting insights into processes associated with treeline dynamics.

Climate variables explored were: mean annual temperature (*AnnualTemp)*, mean winter (December, January, February) temperature (*WinterTemp)*, mean summer (June, July, August) temperature (*SummerTemp)*, summer minimum temperature (*SummerTemp_min*), mean annual precipitation (*AnnualPrecip*), mean winter precipitation (*WinterPrecip)*, precipitation as snow (mm) between August in previous year and July in current year (*PAS*), mean summer precipitation (*SummerPrecip*), summer heat-moisture index (*SHM*), growing degree days >5 °C (*DD5*), and the number of frost-free days (*NFFD*). The variables were calculated as the difference between the repeat and the historic image, with positive values indicating an increase over the period of interest.

### Statistical analyses

We used generalized linear mixed models (GLMM) to determine if treeline ecotone advance was explained by a variety of ecological, climatic, and site variables. The response variable used was whether or not treeline advance was detected between the historic and repeat images (0 = stable/retreat, 1 = advance), which we analyzed using the “binominal” family (‘lme4’ package^28^) in program R^29^. For this analysis, coding retreating treelines as ‘0′ will potentially bias our results towards detection of advancing treelines. However, retreating treelines represent 1–2% of all sites examined and thus will not have a significant impact on our interpretation of the results. To explain treeline advance, we used a variety of categorical and continuous variables in the initial models, all of which have been used elsewhere to explain treeline movement e.g.^12^. The categorical explanatory variables used were: *Disturbance* (0/1/2) and *Aspect* (warm/cold). The continuous explanatory variables used were: *Slope* (°) of modern treeline, time span between photos (*Span*, years), *Elevation* (m), the latitude of each treeline (*Latitude*), and the change in climate variables described above. The variable *MountainID* was created by giving each mountain used in this study a unique identifier as there were some cases of multiple photographs of different treelines on the same mountain. We included *MountainID* as a random effect in our model. Continuous predictors were mean-centered and scaled by 1 standard deviation (SD). P-values for model variables were calculated using Kenward-Roger standard errors and degrees of freedom (df).

To explore the factors influencing treeline advance, we included all explanatory variables including the random effect. Prior to fitting models, we tested for collinearity of explanatory variables and removed those variables that had Pearson’s product moment correlation values greater than 0.60 and −0.60.

## Results

### Quantifying treeline change

Over a mean time period of 93.52 ± 1.20 years, we examined a combined total of 197 km of treeline ecotones across 5 degrees of latitude in the Canadian Rocky Mountains. There were 13 disturbances documented in historic images, compared to 7 disturbances documented in repeat images. While not considered a disturbance in this study unless obscuring at least one segment, 30 mass wasting deposits were a prominent feature in both historic and repeat images. Of the 104 treeline ecotones quantified, 22 were classified as abrupt and 82 as diffuse. The mean slope across the treeline ecotone was 25.54 ± 0.76°. With aspect, 42 treelines were classified as warm and 62 were classified as cold. The mean elevation of the treeline ecotone was 2155.36 ± 13.31 m. For climatic variables, annual, winter, and summer air temperatures, when averaged across all treelines, increased over the period of study (+0.815 ± 0.02 °C, +2.07 ± 0.03 °C, +0.02 ± 0.02 °C, respectively; Supplementary Information). A pattern of decrease was observed for annual, winter and summer precipitation (−68.19 ± 8.19 mm, −42.26 ± 1.67 mm, −5.73 ± 3.22 mm, respectively; Supplementary Information).

**Figure 3:**
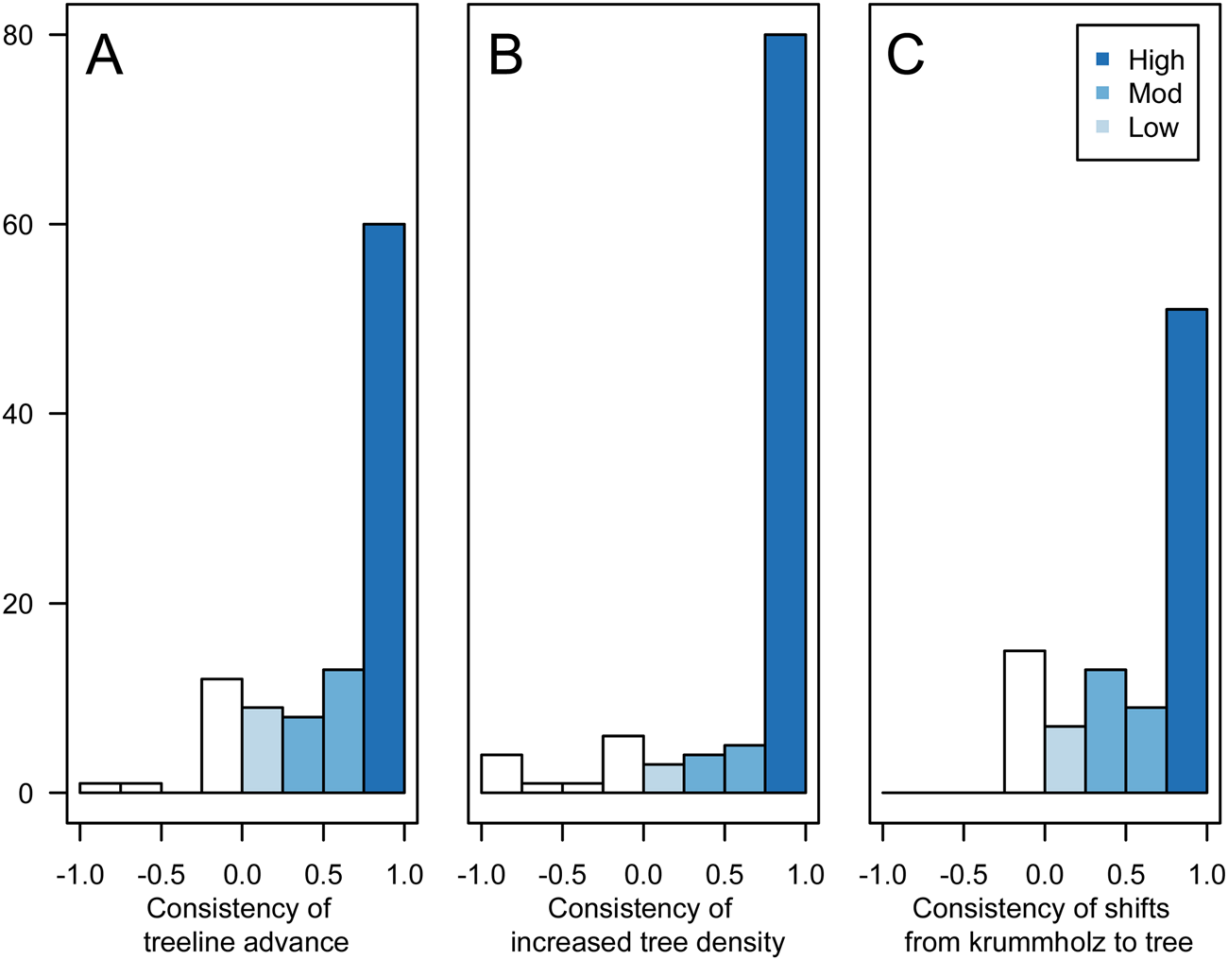
Consistency of treeline advance calculated as the proportion of (**A**) each treeline site reported to be advancing; (**B**) consistency of increased tree density at the treeline calculated as the proportion of each treeline site where local tree density was increasing; and 3) consistency of treeline sites where krummholz were documented to change into trees, determined by the growth of a leader stem. Color gradient from light to dark blue corresponds to consistency scores of Low (0.25–0.49), Moderate (0.5–0.75), and High (1). Values less than or equal to zero are colored white.

**Figure 4:**
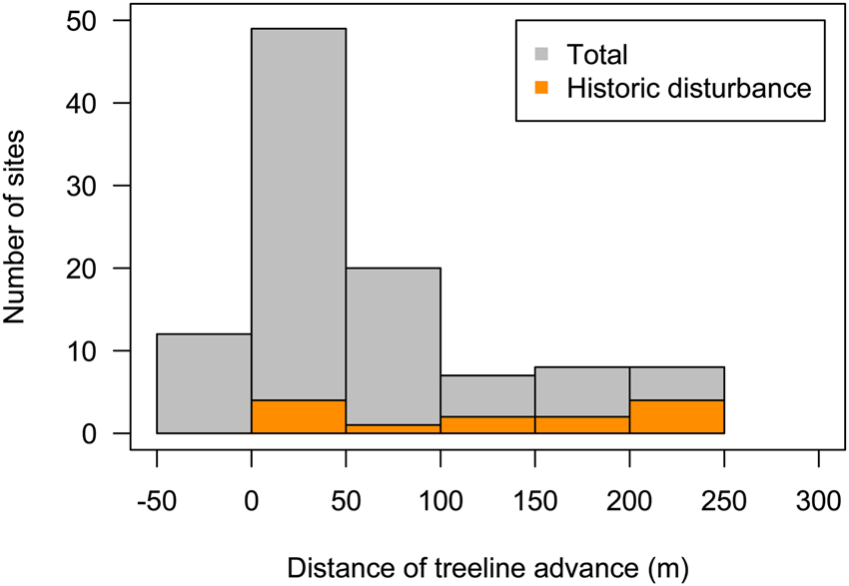
Mean distances of treeline advance between the historic and repeat images from the Canadian Mountain West (n = 104), reported at each site in distances bins. Positive values are representative of sites that experience treeline advance while sites with negative values experienced recession. Sites with evidence of historic disturbance are shown in orange (n = 13).

Overall, 90/104 (87%) of the treelines examined had advanced (Fig. 3A). For treeline advance, only 3 treelines showed overall negative values, indicative of recession, and 11 of the treelines were stable (i.e., no change detected) throughout the study period. For those treelines showing some advance, the consistency of treeline advance at the site level was low (0.25) for 9 treelines while 21 treelines had moderate consistency (0.5–0.75). High consistency of treeline advance (1), was documented at 60 treelines (Fig. 3A). The mean distance of treeline advance at each site varied considerably with a sizable proportion of large advances (i.e., > 100 m) associated with historic disturbances, relative to the number of documented disturbances (Fig. 4).

Tree density increased at 93/104 (89%) of the treeline ecotones (Fig. 3B). Six treeline ecotones experienced a decrease in tree density over the study period and 5 treeline ecotones had no detectable differences in tree density. For treeline ecotones that had at least 1 segment with increased tree density, the consistency was low (0.25) for 3 treeline ecotones while 21 treeline ecotones had moderate consistency (0.5–0.75). High consistency (1) of increased tree density was documented across 80 treeline ecotones (Fig. 3B).

Shifts in growth form from krummholz to trees were observed at 79/95 (83%) of the treelines (Fig. 3C). Fifteen treeline ecotones experienced no detectable changes in growth form from krummholz to trees. For treeline ecotones that had at least 1 segment with detectable shifts of krummholz to tree growth forms, the consistency was low (0.25–0.49) for 9 treeline ecotones while 20 treeline ecotones had moderate consistency (0.5–0.75). High consistency (1) of growth form shifts was documented across 50 treeline ecotones (Fig. 3C). Shifts in growth form were unable to be documented for 9 treeline ecotones due to the quality of the images.

### Factors explaining treeline change

**Figure 5:**
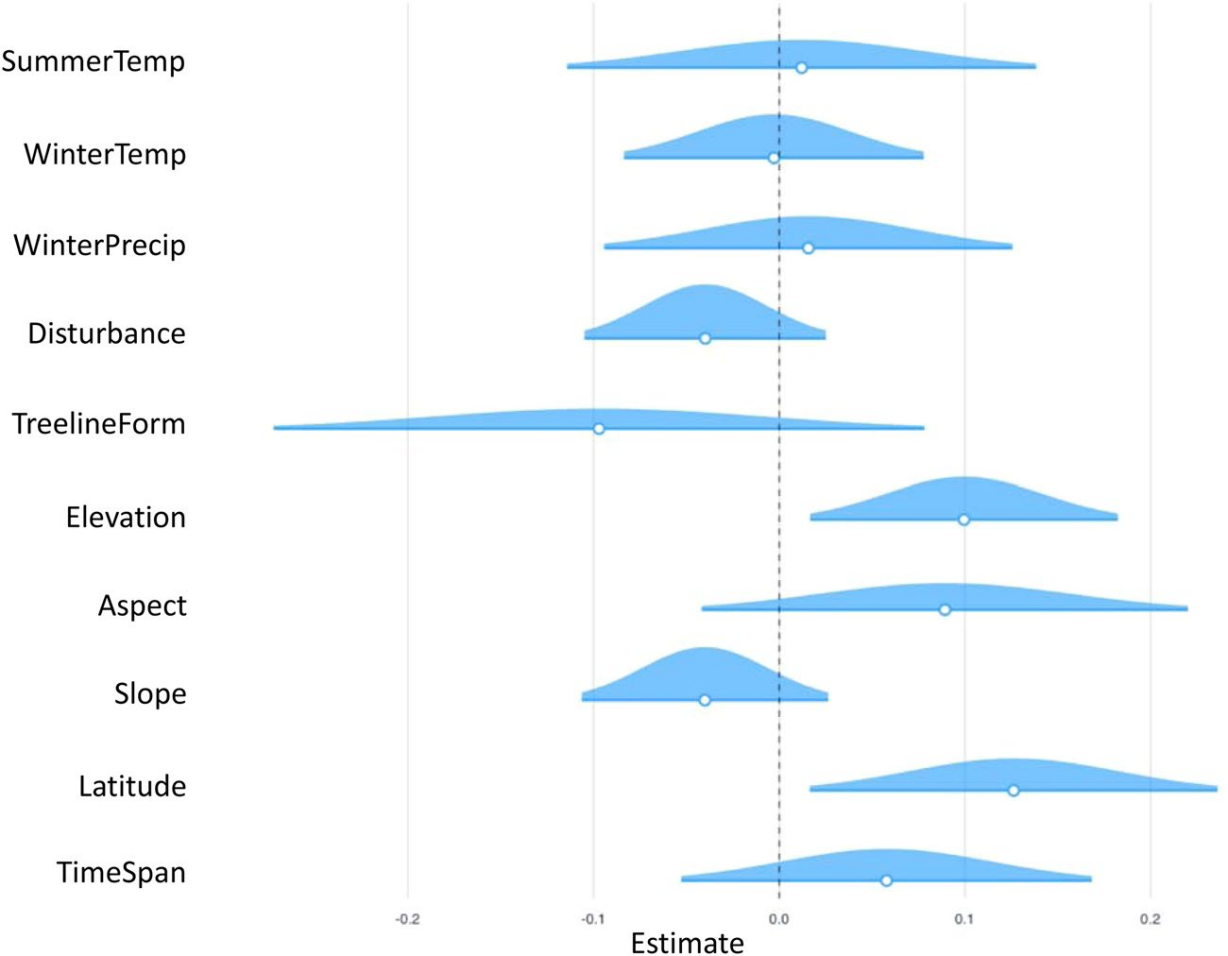
Results from generalized linear mixed effects model showing the mean, distributions, and 95% credible intervals for the parameter estimates showing the effect of each explanatory variable on the probability of treeline advance with mountain location as the random effect. Credible intervals overlapping the zero line are considered not to be significant.

The advance of treelines between the historic and repeat images were best explained by the elevation of treeline and the latitude of the treeline, based on credibility confidence intervals that don’t overlap with zero and with significant p-values (Table 1; Fig. 5). Treelines at higher latitudes (ß = 0.253  ±0.113, p-value = 0.014) and at higher elevation (ß = 0.199 ±0.087, p-value = 0.012) had a significantly greater probability of advancing. There was also evidence of microclimatic (i.e., warm aspects) control on the position of the treeline ecotone (ß = 0.089 ±0.069, p-value = 0.099).

**Table 1:** Results from generalized linear mixed effects model (GLM) for treeline response (0/1) over the last century. Significance values are **p < 0.05; *p = 0.05–0.1.

	Estimate (ß)	SE	t-value	df	p-value
(Intercept)	0.910	0.088	10.373	92	<0.001^**^
TimeSpan	0.116	0.114	1.018	65	0.156
Latitude	0.253	0.113	2.235	82	0.014^**^
Slope	−0.080	0.069	−1.159	92	0.125
Aspect	0.089	0.069	1.298	82	0.099^*^
Elevation	0.199	0.087	2.295	91	0.012^**^
TreelineForm	−0.097	0.091	−1.071	90	0.143
Disturbance	−0.069	0.059	−1.179	92	0.121
WinterPrecip	0.031	0.114	0.276	78	0.391
WinterTemp	−0.006	0.084	−0.068	84	0.473
SummerPrecip	0.024	0.131	0.185	86	0.427

A number of explanatory variables, including disturbance (fire and insects) and all the climatic variables in the model, were not significantly related to treeline advance as the credibility confidence intervals overlap with zero with non-significant p-values (Fig. 5). The explanatory climate variables *AnnualPrecip, SummerTemp_min, AnnualPrecip, PAS, SummerPrecip, SHM, DD5, and NFFD* were not included in the final model due to high collinearity with other explanatory variables.

## Discussion

In our quantitative analysis of treeline ecotone change in the Canadian Rocky Mountains, we found strong evidence of broad landscape change over the past century with most sites having experienced an advance of the treeline and increased tree density at the treeline ecotone.

While high variability of treeline change over the last century is commonly reported in the literature e.g.^12^, we found evidence of high elevation ecosystem change at most sites, further supported by a highly consistent response at the site level. Within our study area, a similar pattern was documented by Luckman & Kavanagh^30^ where there was local variability in treeline change. The mechanisms by which the position of treeline shifts upward rely first on the production of viable seeds, suitable seedbed for these seeds, and ultimately the establishment of these seedlings into saplings and trees. At broad spatial scales and with no interannual resolution, viable seed production and establishment on suitable seedbed is not detectable using our methods.

However, the first detectable changes would arise from the local dispersal of seeds into areas that offer some protection from environmental factors. This infilling of trees at the treeline ecotone was observed at nearly every site, with high consistency across each site. In the absence of disturbance, this trend has been widely observed^12^, coinciding with warm temperatures over the past few decades and may result in lower than expected treeline advance due to increased competition^31^. The second detectable change is the shift in the upper extent of where trees are growing. These advances in treeline were reported at nearly as many sites as saw increases in tree density, however, at the site level, this response was less uniform. High levels of variability in treeline advance that are reported in the literature are attributed to biotic factors as well as recent changes in climate^32^. Lastly, treeline can also advance through a shift in growth form from prostrate krummholz to erect trees, a process that does not require the establishment of new individuals. The majority of our sites (83%) showed evidence of this shift in growth form, likely attributed to individuals taking advantage of the favorable climate conditions. Luckman *et al.*^33^, described a similar trend around the Athabasca and Dome Glaciers at a south facing site.

We found that treelines at higher latitudes and those at higher elevation were more likely to have advanced over the last century. Our most likely explanation of this finding is that the impacts of climate change have been more pronounced at higher latitudes and higher elevations. However, the climate data included in our final model did not support this narrative. More southern latitudes experienced a greater extent of winter warming with no clear trend for summer and annual temperature data (Supplementary Information). Precipitation data may not be fully representative of areas with high topographical variation, but for all sites, the overall trends associate decreased winter precipitation with treeline advance. The clustering of sites, related to resampling efforts, means that we don’t have continuous representation across the latitudinal gradient which could potentially obscure our results. Other explanations for the pattern of sites at higher latitude and elevation showing more advance are that they are under stronger climatic control than sites at lower latitudes and elevations. Unfortunately, we do not have data for specific drivers at this scale. Non-climatic controls on treeline position in the southern Canadian Rocky Mountains support this explanation^34^, but it is not possible to scale these results across the spatial extent of our study area.

As expected, our findings support generally observed patterns of treelines being more likely to advance when on more gradual slopes and on warmer aspects. Gentle slopes may make it easier for seeds to disperse greater distances^35^ and may experience greater retention of snow^36,37^. Deeper snow has been attributed to decreased winter abrasion, especially important for enhanced survival at early life history stages – a key component of treeline advance^38^. Our approach may have resulted in higher levels of treeline change detection at sites with gradual slopes given that an upslope displacement would have a larger horizontal displacement. Warmer aspects generally lose snow earlier in the spring, giving trees an advantage throughout the short growing season. These findings are supported by other studies, most notably Danby & Hik^14^ and Luckman & Kavanagh^30^ who show that treeline advance occurred on warm aspects, while cool aspects did not support advancing treelines. Warm aspects are also more likely to have enhanced permafrost thaw, or absence of permafrost, potentially leading to greater ecosystem change^39^.

Treeline form is often cited as a significant predictor of treeline advance, but we did not find it to be a strong predictor of treeline change in the Canadian Rocky Mountains. We observed general patterns of both abrupt and diffuse treeline forms being associated with advance with a tendency of abrupt treelines (21 of 22) exhibiting a higher likelihood of advance compared to diffuse treelines (69 of 82). We did not differentiate between all possible categories of treeline form as described by Harsch & Bader^40^ because of methodological limitations. This pooling of treeline forms may have led to contrasting conclusions. However, given the large number of responding treelines, diffuse and abrupt, documented in our study, we suggest that treeline form may be less important than other factors over the time period examined and the extent of change observed.

While disturbance was not a significant determinant of treeline ecotone change, there were visual associations between disturbance and the magnitude of treeline response. Treelines that advanced significant distances over the past century (>100 m), were in some cases associated with historic disturbance events. Another study using repeat photography to quantify treeline change over the 20^th^ century in Scandinavia, found that even with significant climate warming, disturbance, vis-à-vis reindeer grazing, was the best predictor of whether treeline advance would occur^41^. The type, frequency, severity and extent of these disturbances were not the focus of this study but would be amenable to analysis using similar methods. While we did not differentiate between disturbance types, as we were unable to ground truth all sites, we presumed that high elevation areas of tree mortality were attributed to past fire, though they could be related to outbreaks of mountain pine beetle (*Dendroctonus ponderosae*) or possibily spruce bark beetle (*Dendroctonus rufipennis*) or balsam bark beetle (*Dryocoetes confusus*). Despite the increase in burned forest area across Canada over the last half century, the trends in the Canadian Rocky Mountains are less sure^42^. Within the southern Canadian Rocky Mountains, the fire-regime in the subalpine, in terms of median fire return interval, is within the historic range of variation at 121 years^43^. Our data generally support these patterns of disturbance being an important factor in the Canadian Rocky Mountains over the past century with 13 historic disturbances and 7 disturbances detected in the repeated images, likely attributed, in part, to fire suppression in much of the study area throughout the past century. Recent work in the northern Canadian Rocky Mountains by Higuera *et al.*^44^ shows a strengthening fire-climate relationship between 1985–2008.

The extent of change that we document in this study was surprising given that broad scale detection of treeline advance, such as we were using in our study, focuses on the position of adult trees and does not consider regeneration. Thus, we are presenting conservative estimates of treeline change as sites experiencing recent increases in seedlings and saplings would be undetected by our methods, especially given the slow growth rates of seedlings and saplings observed at treeline^30^. We also acknowledge the importance of species responding differentially to changes in climate. Interactions within and between species were not possible to include in our analyses, again because of the broad spatial scale we used to detect change. Without these early life history and species-specific data, we can make inferences about important factors driving ecosystem change but specific mechanisms remain the topic of future studies.

The use of historic and repeat oblique images provides important references by which to demonstrate ecosystem change. Using a more constrained temporal period of 17–20 years, Butler *et al.*^45^ found that the majority of treelines examined in the US Rocky Mountains appeared to be stable, thus reinforcing the importance of historic references. The scope, coverage, and resolution of the Mountain Legacy Project collection covers almost all of the western Canadian cordillera–a vast region including the Canadian Rocky Mountains in Alberta and British Columbia (the focus of our study). High resolution oblique photographs also offer advantages in viewing steep terrain from angles more appropriate to analyze high elevation mountain regions. Using high resolution, comprehensive and systematic oblique images such as those found in the Mountain Legacy Project collections in combination with custom software holds great potential for providing new insight into the dynamics of change in rapidly-shifting high altitude and latitude ecosystems.

## Supplementary information

Supplementary Information.
