# Publisher Correction: A century of high elevation ecosystem change in the Canadian Rocky Mountains

**DOI:** 10.1038/s41598-020-76735-6

**Published:** 2020-11-10

**Authors:** Andrew Trant, Eric Higgs, Brian M. Starzomski

**Affiliations:** 1 School of Environment, Resources and Sustainability, University of Waterloo, Waterloo, ON Canada; 2 School of Environmental Studies, University of Victoria, Victoria, BC Canada

Correction to: *Scientific Reports* 10.1038/s41598-020-66277-2, published online 16 June 2020

This Article contains typographical errors in the Acknowledgements section.

“We appreciate key insights and software developments provided by Mary Sanseverino, Mike Whitney, Beatrice Proudfoot, Jodi Axelson, Julie Fortin, Dan Shugar to the list of people acknowledged in between Julie Fortin and Frederic Jean Frédéric Jean, and Jason Fisher.”

should read:

“We appreciate key insights and software developments provided by Mary Sanseverino, Mike Whitney, Beatrice Proudfoot, Jodi Axelson, Julie Fortin, Dan Shugar, Frédéric Jean, and Jason Fisher.”

