# Supplementary material for: A century of high elevation ecosystem change in the Canadian Rocky Mountains
Source: Sci Rep. 2020 Jun 16;10:9698. doi: 10.1038/s41598-020-66277-2 (PMC7298051; doi:10.1038/s41598-020-66277-2)
Supplement: Supplementary file 1 — Supplementary Information. [file 41598_2020_66277_MOESM1_ESM.pdf]

Supplementary Information

A century of high elevation ecosystem change in the Canadian Rocky Mountains

Andrew Trant, Eric Higgs, and Brian M. Starzomski

Supplementary Information

A century of high elevation ecosystem change in the Canadian Rocky Mountains

SITE LOCATIONS

PhotoPairID	HistoricYear	RepeatYear	Location_SurveyStation	Latitude	Longitude
1	1913	2008	Stn. 27 Dutch Creek Head No. 1; Crowsnest Forest Reserve and Waterton Lakes National Park	49.945886	-114.682723
2	1913	2008	Stn. 27 Dutch Creek Head No. 1; Crowsnest Forest Reserve and Waterton Lakes National Park	49.940655	-114.663105
3	1913	2008	Stn. 27 Dutch Creek Head No. 1; Crowsnest Forest Reserve and Waterton Lakes National Park	49.925053	-114.649325
4	1913	2008	Stn. 27 Dutch Creek Head No. 1; Crowsnest Forest Reserve and Waterton Lakes National Park	49.914612	-114.639623
8	1913	2008	Stn. 43 Grassy Ridge; Crowsnest Forest Reserve and Waterton Lakes National Park	49.927874	-114.621568
8	1913	2008	Stn. 43 Grassy Ridge; Crowsnest Forest Reserve and Waterton Lakes National Park	49.927874	-114.621568
9	1913	2008	Stn. 43 Grassy Ridge; Crowsnest Forest Reserve and Waterton Lakes National Park	49.941069	-114.605155
11	1913	2008	Stn. 54 Sentinel Pass West No. 2; Crowsnest Forest Reserve and Waterton Lakes National Park	50.217702	-114.468354
11	1913	2008	Stn. 54 Sentinel Pass West No. 2; Crowsnest Forest Reserve and Waterton Lakes National Park	50.217702	-114.468354
12	1913	2008	Stn. 54 Sentinel Pass West No. 2; Crowsnest Forest Reserve and Waterton Lakes National Park	50.248445	-114.481374
13	1913	2008	Stn. 54 Sentinel Pass West No. 2; Crowsnest Forest Reserve and Waterton Lakes National Park	50.232963	-114.468565
14	1913	2008	Stn. 58 Willow Creek No. 2; Crowsnest Forest Reserve and Waterton Lakes National Park	50.197743	-114.419133
19	1913	2008	Stn. 12 Boundary No. 2A; Crowsnest Forest Reserve and Waterton Lakes National Park	50.129344	-114.713131
24	1919	2009	Stn. 235; Bow-Clearwater Forest Reserve	51.788269	-115.688667
25	1919	2009	Stn. 235; Bow-Clearwater Forest Reserve	51.804733	-115.728716
25	1919	2009	Stn. 235; Bow-Clearwater Forest Reserve	51.804733	-115.728716
28	1927	2011	Stn. 443; Brazeau Forest Reserve and Jasper National Park	52.092321	-116.628981
29	1927	2011	Stn. 443; Brazeau Forest Reserve and Jasper National Park	52.051207	-116.560260
30	1927	2011	Stn. 443; Brazeau Forest Reserve and Jasper National Park	51.988157	-116.616317
36	1927	2011	Stn. 444; Brazeau Forest Reserve and Jasper National Park	52.016475	-116.751307
36	1927	2011	Stn. 444; Brazeau Forest Reserve and Jasper National Park	52.024700	-116.733976
36	1927	2011	Stn. 444; Brazeau Forest Reserve and Jasper National Park	52.024700	-116.733976
37	1927	2011	Stn. 446; Brazeau Forest Reserve and Jasper National Park	51.947946	-116.842435
37	1927	2011	Stn. 446; Brazeau Forest Reserve and Jasper National Park	51.961592	-116.830190
38	1927	2011	Stn. 446; Brazeau Forest Reserve and Jasper National Park	52.028255	-116.705458
39	1927	2011	Stn. 446; Brazeau Forest Reserve and Jasper National Park	52.025924	-116.736925
39	1927	2011	Stn. 446; Brazeau Forest Reserve and Jasper National Park	52.031785	-116.716131
42	1927	2011	Stn. 445; Brazeau Forest Reserve and Jasper National Park	52.002345	-116.717093

42	1927	2011	Stn. 445; Brazeau Forest Reserve and Jasper National Park	52.010462	-116.716632
43	1896	2008	Moose Mt Centre; Canadian Irrigation Survey	50.937584	-114.849015
56	1924	2011	Stn. 289 - Casket : Coffin Mtn; Willmore-Kakwa	53.843804	-119.940133
56	1924	2011	Stn. 289 - Casket : Coffin Mtn; Willmore-Kakwa	53.853207	-119.918229
57	1924	2011	Stn. 289 - Casket : Coffin Mtn; Willmore-Kakwa	53.803578	-119.862915
57	1924	2011	Stn. 289 - Casket : Coffin Mtn; Willmore-Kakwa	53.803578	-119.862915
58	1924	2011	Stn. 289 - Casket : Coffin Mtn; Willmore-Kakwa	53.769996	-119.897697
59	1924	2011	Stn. 289 - Casket : Coffin Mtn; Willmore-Kakwa	53.735404	-119.969696
61	1919	2009	Stn. 258	51.785254	-115.871490
61	1919	2009	Stn. 258	51.796400	-115.884421
62	1919	2009	Stn. 258	51.784429	-115.975408
63	1919	2009	Stn. 258	51.797541	-115.989037
64	1919	2009	Clearwater County, AB	51.804733	-115.883387
65	1919	2009	Stn. 265	51.864444	-116.005556
66	1919	2009	Stn. 265	51.858168	-116.061820
67	1919	2009	Stn. 265	51.866371	-115.974464
68	1919	2009	Stn. 265	51.866371	-115.974464
68	1919	2009	Stn. 265	51.866371	-115.974464
69	1927	2009	Stn. 428	51.938597	-116.507876
70	1927	2009	Stn. 428	51.916754	-116.562553
71	1927	2009	Stn. 428	51.935420	-116.557237
72	1927	2007	Stn. 8	53.377304	-118.62959
73	1927	2007	Stn. 8	53.427320	-118.598834
74	1927	2007	Stn. 8	53.439883	-118.652896
75	1927	2007	Stn. 8	53.392178	-118.672215
75	1927	2007	Stn. 8	53.404551	-118.683887
77	1944	2012	Stn. 13	53.590183	-118.393848
78	1914	2004	Stn. 90 Cameron South	49.063819	-114.046051
79	1914	2004	Stn. 90 Cameron South	49.081105	-114.029731
80	1914	2004	Stn. 90 Cameron South	49.055561	-113.973927
80	1914	2004	Stn. 90 Cameron South	49.061594	-113.963479
81	1914	2004	Stn. 90 Cameron South	49.045724	-113.960387
82	1914	2004	Stn. 110 Ruby Ridge No. 2	49.063237	-113.980970

83	1914	2004	Stn. 110 Ruby Ridge No. 2	49.086892	-113.988238
84	1914	2004	Stn. 110 Ruby Ridge No. 2	49.128657	-113.996964
84	1914	2004	Stn. 110 Ruby Ridge No. 2	49.140763	-114.002358
85	1915	2008	Alexander Summit East	49.879422	-114.692894
86	1915	2008	Alexander Summit East	49.873510	-114.679549
87	1915	2008	Alexander Summit East	49.845072	-114.661380
88	1915	2008	Alexander Summit East	49.840211	-114.684529
89	1927	2007	Stn. 36	53.481115	-119.140001
90	1927	2007	Stn. 36	53.484360	-119.111352
91	1927	2007	Stn. 36	53.474990	-119.090875
93	1927	2007	Stn. 51	53.517091	-119.191161
95	1924	2011	Stn. 292 - Sheep Creek South	53.795669	-119.823088
96	1914	2006	Stn. 19 Mount Coulthard No. 1	49.546333	-114.589593
97	1914	2006	Stn. 19 Mount Coulthard No. 1	49.577597	-114.571155
98	1914	2006	Stn. 19 Mount Coulthard No. 1	49.544344	-114.568363
99	1914	2006	Stn. 21 Link Creek West No.1	49.535375	-114.564790
100	1914	2006	Stn. 21 Link Creek West No.1	49.550912	-114.553627
101	1914	2006	Stn. 21 Link Creek West No.1	49.541226	-114.568234
102	1915	2005	Province South Divide	49.189898	-114.300060
103	1915	2005	Province South Divide	49.183044	-114.342175
104	1924	2011	Stn. 292 - Sheep Creek South	53.910283	-119.884418
105	1924	2011	Stn. 292 - Sheep Creek South	53.817069	-119.807081
106	1888	2009	Mt. Alymer East	51.283037	-115.327238
106	1888	2009	Mt. Alymer East	51.283037	-115.327238
108	1888	2009	Mt. Alymer East	51.249888	-115.393149
108	1888	2009	Mt. Alymer East	51.258251	-115.427875
110	1888	2009	Mt. Alymer East	51.325073	-115.348701
110	1888	2009	Mt. Alymer East	51.325073	-115.348701
111	1888	2009	Mt. Alymer East	51.299754	-115.348798
111	1888	2009	Mt. Alymer East	51.321806	-115.334327
113	1888	2009	Mt. Costigan West	51.269620	-115.283515
118	1889	2014	Grotto Mt.	51.119018	-115.278583
118	1889	2014	Grotto Mt.	51.131639	-115.294564

119	1890	2001	Grotto Mt.	51.115143	-115.312800
119	1890	2001	Grotto Mt.	51.115143	-115.312800
119	1890	2001	Grotto Mt.	51.123089	-115.310771
120	1905	2009	Wedge Mt East (C4)	50.847294	-115.132013
120	1905	2009	Wedge Mt East (C4)	50.851380	-115.132298
121	1905	2009	Wedge Mt East (C4)	50.854379	-115.133358
122	1905	2009	Wedge Mt East (C4)	50.814294	-115.109829
123	1905	2009	Wedge Mt East (C4)	50.846652	-115.109743
123	1905	2009	Wedge Mt East (C4)	50.846652	-115.109743
127	1914	2004	Stn. 103 Sheep Mountain (Vimy)	49.037224	-113.868118

A century of high elevation ecosystem change in the Canadian Rocky Mountains

SITE VARIABLES

PhotoPairID	Elev	Slope	AspectWC	TreelineForm	DisturbanceRH	TreelineAdvanceConsistency	DensityConsistency	KrummholzConsistency
1	2087	20.78	C	A	0	1	1	1
2	2262	18.73	W	A	0	0.5	0.75	1
3	2203	20.01	W	A	0	1	1	1
4	2190	20.09	W	A	0	0.75	0.75	0.5
8	2200	18.91	W	A	0	1	1	1
8	2263	21.49	C	A	0	0.66	1	1
9	2271	14.93	W	A	0	1	1	1
11	2253	34.80	W	D	0	1	1	0.75
11	2253	26.09	W	D	0	1	1	1
12	2231	18.13	W	D	0	1	1	1
13	2002	24.55	C	D	0	1	1	0.75
14	2027	28.82	C	D	H	0	1	1
19	2189	11.34	C	D	0	0	1	1
24	2245	19.37	C	D	0	1	1	1
25	2270	23.69	W	D	0	1	1	1
25	2270	12.28	C	D	0	1	1	1
28	2292	39.77	C	D	0	0	1	1
29	2279	34.68	W	D	R	0	0	1
30	1744	46.53	C	A	R	0.25	0.25	1
36	2127	15.49	C	D	0	1	1	1
36	2194	31.19	W	D	H	1	1	0.33
36	2194	13.34	C	D	0	1	1	1
37	2268	25.89	W	D	0	1	1	1
37	2018	29.32	C	D	H	0	1	NA
38	2271	33.24	W	D	H	1	1	1
39	2200	32.32	W	D	H	1	1	1
39	2230	20.65	W	D	H	1	1	1
42	2171	35.09	W	D	H	0.75	1	1

42	2108	27.96	C	D	H	0.25	0	0.25
43	2172	20.18	C	D	0	1	1	1
56	1869	12.36	W	D	R	1	1	1
56	2003	19.84	W	A	0	0.25	1	1
57	1917	24.72	C	A	0	1	1	1
57	1917	28.73	C	A	0	0.75	1	1
58	1918	19.02	C	D	0	1	1	1
59	1834	14.51	C	A	0	0.25	1	1
61	2207	25.81	C	D	0	0.5	1	0
61	2309	20.10	W	D	0	1	1	0
62	2325	30.77	W	D	0	1	1	1
63	2317	15.20	C	D	0	1	1	1
64	2412	26.95	W	D	0	1	1	1
65	2046	38.54	C	A	0	0.25	0	0.5
66	2319	18.95	C	D	0	1	1	1
67	2288	30.69	C	D	0	1	0	0
68	2089	34.60	C	D	0	0	1	0
68	2089	25.53	W	D	0	1	1	1
69	2279	25.98	W	D	0	0.5	1	1
70	2205	33.77	W	D	0	0.75	1	1
71	2285	39.36	W	D	0	1	1	0.5
72	1990	14.42	W	D	0	0.25	1	1
73	2164	25.24	W	D	0	1	1	1
74	2186	40.55	W	D	0	1	1	1
75	1951	12.58	C	D	0	1	1	0.66
75	2183	7.16	W	D	0	1	1	1
77	2006	17.87	W	A	0	0.75	0.5	0.5
78	2341	40.00	W	D	0	0.25	0.5	0.33
79	2058	35.29	W	D	0	1	1	0.66
80	2119	31.57	W	D	0	0	-0.5	0
80	2280	35.23	W	D	0	0	0	0
81	1914	27.47	C	D	0	0	0	NA
82	1915	42.15	C	D	0	-1	-1	0

83	2226	17.86	W	A	0	0	1	0
84	1797	27.94	W	D	0	1	1	NA
84	2020	21.09	W	D	0	1	0.25	1
85	2248	35.79	W	D	0	1	1	0.25
86	2265	22.27	W	D	0	1	1	0.25
87	2211	26.79	W	D	0	1	1	0.5
88	2160	32.39	C	A	0	0.5	1	0.5
89	2200	28.64	W	A	0	0.5	1	0.5
90	2227	33.50	W	A	0	0.75	0.75	0.75
91	2136	39.16	W	A	0	1	1	NA
93	2013	23.73	W	A	0	0.75	0.75	NA
95	1996	31.31	C	D	0	1	1	1
96	2228	32.68	W	D	0	1	1	0
97	2109	26.08	W	D	0	0	-0.75	0
98	2098	32.44	C	D	0	1	0.25	NA
99	2221	19.85	C	D	R	0.5	-0.75	0.5
100	1896	23.51	W	D	R	-0.5	-1	0.5
101	2087	27.54	C	D	0	0.75	-0.25	1
102	2275	17.60	W	D	0	0.25	0.5	0.25
103	2094	17.54	W	D	0	0.25	1	0
104	2064	18.52	W	D	R	1	1	0
105	1952	23.15	W	A	R	0.75	0.75	0.25
106	2224	27.04	C	D	H	1	1	0
106	2147	24.97	W	D	H	1	1	1
108	2093	32.41	C	D	0	1	1	NA
108	1979	36.93	C	A	0	1	1	NA
110	2252	22.40	W	D	0	1	1	1
110	1789	21.13	W	D	0	0	1	NA
111	2222	25.37	W	D	0	1	1	1
111	2210	22.39	W	D	H	1	1	1
113	2111	25.38	C	D	0	0.5	1	0.75
118	2104	29.40	W	D	0	0.75	0.5	0.5
118	2295	27.37	W	D	0	0.75	1	0

119	2216	26.06	W	D	0	1	1	0.25
119	2215	18.41	W	D	0	1	1	1
119	2341	23.05	C	D	0	0.75	1	0.25
120	2448	26.40	C	D	0	1	1	1
120	2430	24.13	C	D	0	1	1	0
121	2375	25.13	C	D	0	1	1	0.75
122	2235	16.45	C	D	H	1	1	0
123	2309	22.27	W	D	H	1	1	0.5
123	2255	20.42	C	D	0	1	1	0.75
127	2165	25.84	W	D	0	0.5	1	0.75

A century of high elevation ecosystem change in the Canadian Rocky Mountains

CLIMATE VARIABLES

PhotoPairID	MATdiff	MAPdiff	SHMdiff	DD5diff	NFFDdiff	PASdiff	Tmin_sm_diff	Tave_wt_diff	Tave_sm_diff	PPT_wt_diff	PPT_sm_diff
1	0.9	-40	0.7	52	9	-52	0.7	2	0.3	-34	-3
2	0.9	-43	0.6	50	10	-56	0.7	2.1	0.3	-38	-3
3	1	-36	0.6	54	10	-51	0.7	2.1	0.3	-33	-2
4	0.9	-33	0.5	56	10	-51	0.7	2.1	0.3	-32	-1
8	0.9	-31	0.5	55	9	-49	0.7	2.1	0.4	-30	-1
8	0.9	-33	0.4	54	9	-51	0.7	2.1	0.3	-31	-1
9	0.9	-31	0.3	53	10	-49	0.6	2.1	0.3	-30	0
11	0.9	-46	0.7	33	8	-54	0.6	2.1	0.1	-28	-5
11	0.9	-46	0.7	33	8	-54	0.6	2.1	0.1	-28	-5
12	0.8	-49	0.8	31	8	-55	0.6	2.1	0.1	-29	-4
13	0.8	-40	0.6	36	8	-49	0.6	2.1	0.1	-24	-4
14	0.9	-35	0.4	37	8	-46	0.6	2.1	0.2	-22	-3
19	0.9	-59	1.1	36	10	-61	0.6	2	0.2	-41	-9
24	0.8	-194	2.5	12	12	-122	0.8	2.2	0	-50	-60
25	0.8	-199	2.4	12	13	-126	0.8	2.2	-0.1	-53	-60
25	0.8	-199	2.4	12	13	-126	0.8	2.2	-0.1	-53	-60
28	0.9	-152	1.8	22	14	-133	0.9	2.3	0.1	-68	-25
29	0.8	-152	2.1	23	14	-128	0.9	2.3	0.1	-65	-27
30	0.9	-109	3.1	29	13	-89	0.9	2.3	0.1	-46	-20
36	0.8	-138	2.5	24	14	-124	0.8	2.3	0.2	-67	-19
36	0.9	-149	2.4	22	14	-134	0.9	2.2	0.1	-72	-21
36	0.9	-149	2.4	22	14	-134	0.9	2.2	0.1	-72	-21
37	0.9	-143	2	33	12	-134	0.9	2.3	0.1	-74	-15
37	0.9	-124	2.2	34	12	-115	0.9	2.3	0.1	-64	-14
38	0.8	-164	2.2	22	13	-146	0.9	2.3	0.1	-78	-23
39	0.9	-149	2.3	22	14	-134	0.9	2.3	0.2	-72	-20
39	0.9	-158	2.3	22	13	-142	0.9	2.3	0.1	-76	-22
42	0.9	-149	2.4	23	14	-133	0.9	2.2	0.2	-71	-21

42	0.9	-145	2.3	23	13	-128	0.9	2.3	0.2	-69	-21
43	0.7	-87	1.9	13	7	-64	0.6	2.1	0	-29	-17
56	0.8	42	-1	39	13	-40	0.9	2.2	0.2	-26	53
56	0.8	35	-1.1	36	13	-33	0.9	2.2	0.2	-23	47
57	0.8	44	-1	39	12	-42	0.8	2.2	0.3	-27	57
57	0.8	44	-1	39	12	-42	0.8	2.2	0.3	-27	57
58	0.8	57	-1	38	12	-35	0.9	2.1	0.2	-25	62
59	0.8	70	-1.2	41	12	-25	0.9	2.2	0.3	-19	62
61	-0.2	-54	0.2	-17	-2	-31	-0.1	-0.1	-0.2	-18	-15
61	0.8	-213	2.3	9	13	-147	0.8	2.2	0	-61	-60
62	0.7	-228	2.3	10	12	-162	0.9	2.1	0	-70	-60
63	0.8	-225	2.4	10	12	-160	0.8	2.2	0.1	-70	-58
64	0.7	-223	2.2	10	12	-155	0.9	2.1	0	-66	-61
65	0.7	-171	2.8	14	13	-120	0.9	2.2	0.1	-53	-45
66	0.8	-201	2.5	13	12	-145	0.9	2.2	0	-68	-50
67	0.8	-194	2.3	14	13	-136	0.9	2.2	0	-61	-51
68	0.8	-178	2.6	14	12	-126	0.9	2.2	0	-56	-46
68	0.8	-178	2.6	14	12	-126	0.9	2.2	0	-56	-46
69	0.9	-175	2.2	19	14	-144	0.9	2.2	0.1	-72	-31
70	0.9	-166	2.1	18	13	-141	0.8	2.2	0.1	-70	-28
71	0.9	-169	2.1	19	13	-143	0.8	2.2	0.1	-71	-29
72	0.8	22	-1.4	48	11	-36	0.8	2.1	0.3	-25	46
73	0.8	23	-1	43	12	-37	0.8	2.1	0.3	-25	48
74	0.8	18	-1.1	39	12	-38	0.8	2	0.3	-26	46
75	0.8	21	-1.3	43	12	-34	0.8	2	0.4	-24	45
75	0.8	20	-1	40	12	-38	0.8	2	0.3	-26	48
77	0.8	9	-1.1	41	12	-37	0.8	2	0.3	-21	38
78	1	28	0.3	97	11	-53	1	2	0.7	-45	9
79	0.9	25	0.3	98	11	-49	1	2	0.7	-38	8
80	1	22	0.4	102	11	-62	1.1	1.9	0.7	-43	6
80	0.9	22	0.4	102	11	-72	1	2	0.7	-48	6
81	1	21	0.5	107	11	-66	1.1	2	0.7	-37	5
82	1	23	0.5	103	12	-55	1.1	2	0.7	-37	6

83	0.9	23	0.3	102	11	-67	1	2	0.7	-45	7
84	1	16	0.4	103	11	-51	1	2.1	0.7	-31	6
84	1	16	0.4	101	12	-58	1	2	0.7	-38	7
85	0.9	-41	0.6	59	10	-58	0.7	2	0.3	-39	-2
86	1	-41	0.6	60	10	-59	0.7	2.1	0.4	-38	-2
87	1	-38	0.6	62	10	-58	0.8	2.1	0.3	-38	-2
88	1	-37	0.6	63	10	-58	0.8	2.1	0.4	-37	-1
89	0.7	27	-1.2	39	12	-34	0.8	2	0.4	-25	49
90	0.8	27	-1.1	37	12	-34	0.8	2.1	0.4	-24	49
91	0.8	24	-1.1	37	12	-34	0.8	2	0.3	-24	47
93	0.8	26	-1.3	40	11	-33	0.8	2	0.3	-23	45
95	0.8	42	-1	38	12	-46	0.9	2.1	0.2	-30	60
96	1	-21	0.1	81	13	-62	0.9	2	0.6	-39	5
97	1	-16	0.1	83	12	-55	0.8	2	0.5	-33	4
98	1	-16	0.2	85	12	-56	0.9	2.1	0.5	-34	5
99	1	-19	0.1	82	12	-60	0.9	2.1	0.5	-38	5
100	1	-10	0.1	88	12	-48	0.9	2.1	0.5	-27	4
101	1	-16	0.1	85	12	-56	0.9	2	0.5	-33	5
102	1	13	0	102	11	-62	1.1	2	0.7	-40	11
103	1	16	0	100	11	-54	1	2	0.6	-36	11
104	0.8	23	-1.1	35	12	-37	0.9	2.2	0.3	-25	44
105	0.8	34	-1.1	37	13	-40	0.9	2.2	0.3	-25	51
106	0.7	-142	1.6	8	9	-92	0.8	2.1	-0.1	-46	-37
106	0.6	-136	1.7	9	9	-87	0.7	2.1	0	-44	-36
108	0.7	-126	2	10	9	-77	0.7	2.1	0	-42	-33
108	0.7	-122	2.2	11	8	-76	0.8	2	0	-41	-32
110	0.7	-146	1.5	9	9	-95	0.8	2.1	0	-47	-39
110	0.7	-105	2.1	9	10	-65	0.8	2.1	-0.1	-29	-30
111	0.7	-142	1.6	10	10	-92	0.8	2.1	-0.1	-46	-38
111	0.7	-141	1.6	9	10	-91	0.8	2.1	-0.1	-44	-38
113	0.6	-133	1.7	9	9	-84	0.8	2.1	0	-43	-36
118	0.7	-118	1.6	9	9	-80	0.7	2.1	-0.1	-45	-29
118	0.7	-134	1.4	8	8	-91	0.8	2.1	0	-51	-34

119	0.6	-123	1.6	11	9	-83	0.8	2.1	0	-47	-31
119	0.6	-123	1.6	11	9	-82	0.8	2.1	0	-48	-30
119	-0.3	67	-2.4	-43	-3	45	-0.1	-0.3	-0.3	13	25
120	0.7	-107	1.2	13	8	-78	0.6	2	0	-46	-26
120	0.7	-105	1.1	12	8	-76	0.6	2.1	0	-45	-27
121	0.7	-103	1.2	12	8	-74	0.6	2	-0.1	-44	-26
122	0.7	-92	1.2	12	8	-66	0.7	2.1	0	-38	-24
123	0.7	-99	1.2	12	8	-71	0.6	2.1	0	-41	-25
123	0.7	-96	1.2	12	8	-70	0.6	2.1	0	-40	-24
127	1	13	0.5	108	11	-85	1	2	0.7	-46	4

Supplementary Information

A century of high elevation ecosystem change in the Canadian Rocky Mountains

METADATA

SITE LOCATIONS	DETAILS
PhotoPairID	Unique number given to image pair
HistoricYear	Year that the historic image was taken
RepeatYear	Year that the repeat image was taken
Location_SurveyStation	Place name or station name of photo location
Latitude	Latitude of mountain (from Mountain Legacy Project website)
Longitude	Latitude of mountain (from Mountain Legacy Project website)

SITE VARIABLES	DETAILS
Elev	Elevation (in m) of comtempory treeline
Slope	Slope (in m) of comtempory treeline
AspectWC	Aspect of treeline (W=warm, C=cold)
TreelineAdvanceConsistency	Average consistency score of four segment (0=stable/retreat, 1=advance)
DensityConsistency	Average consistency score of four segment (0=stable/retreat, 1=advance)
KrummholzConsistency	Average consistency score of four segment (0=stable/decrease density, 1=increase in density)
TreelineForm	Average consistency score of four segment (0=stable/less trees, 1=more krummholz to trees)
DisturbanceRH	Disturbance documents in R=repeat or H=historic treelines

CLIMATE VARIABLES	DETAILS
MATdiff	Difference in mean annual temperature
MAPdiff	Difference in mean annual precipitation
SHMdiff	Difference in summer heat moisture index
DD5diff	Difference in growing degree days greater than 5C
NFFDdiff	Difference in number of frost free days
PASdiff	Difference in precipitation as snow
Tmin_sm_diff	Difference in summer minimun temperature
Tave_wt_diff	Difference in winter average temperature
Tave_sm_diff	Difference in summer average temperature
PPT_wt_diff	Difference in winter average precipitation
PPT_sm_diff	Difference in summer average precipitation
